# Lateral Lumbar Interbody Fusion for Ossification of the Yellow Ligament in the Lumbar Spine: First Reported Case

**DOI:** 10.1155/2017/3404319

**Published:** 2017-03-02

**Authors:** Kengo Fujii, Tetsuya Abe, Toru Funayama, Hiroshi Noguchi, Keita Nakayama, Kousei Miura, Katsuya Nagashima, Hiroshi Kumagai, Masashi Yamazaki

**Affiliations:** Department of Orthopaedic Surgery, Faculty of Medicine, University of Tsukuba, 1-1-1 Tennodai, Tsukuba, Ibaraki 305-8575, Japan

## Abstract

When ossification of the yellow ligament (OYL) occurs in the lumbar spine and extends to the lateral wall of the spinal canal, facetectomy is required to remove all of the ossified lesion and achieve decompression. Subsequent posterior fixation with interbody fusion will then be necessary to prevent postoperative progression of the ossification and intervertebral instability. The technique of lateral lumbar interbody fusion (LLIF) has recently been introduced. Using this procedure, surgeons can avoid excess blood loss from the extradural venous plexus and detachment of the ossified lesion and the ventral dura mater is avoidable. We present a 55-year-old male patient with OYL at L3/4 and anterior spondylolisthesis of L4 vertebra, with concomitant ossification of the posterior longitudinal ligament, who presented with a severe gait disturbance. He underwent a 2-stage operation without complications: LLIF for L3/4 and L4/5 was performed at the initial surgery, and posterior decompression fixation using pedicle screws from L3 to L5 was performed at the second surgery. His postoperative progress was favorable, and his interbody fusion was deemed successful. Here, we present the first reported case of LLIF for OYL of the lumbar spine. This procedure can be a good option for OYL of the lumbar spine.

## 1. Introduction

Ossification of the yellow ligament (OYL) involves replacement of the yellow ligament by mature lamellar bone. Reportedly, OYL occurs predominantly in the Japanese population, most commonly in the lower thoracic spine [1–3]. The incidence of OYL of the whole spine in the general population is reportedly 3.8% to 36% [1, 3–5]. The incidence of OYL in the lumbar spine is reportedly 8.6% to 11.3%, and it is not rare to see coexistence of lumbar and thoracic OYL [4, 6].

When ossification exists in the lumbar spine and extends to the lateral wall of the spinal canal, facetectomy is required to remove all of the ossified lesion. Posterior lumbar interbody fusion (PLIF), transforaminal lumbar interbody fusion (TLIF), or posterior fixation may be necessary to prevent postoperative progression of the ossification or intervertebral instability [7].

In recent years, the technique of lateral lumbar interbody fusion (LLIF) has been introduced, and it is now performed widely for degenerative lumbar conditions [8–10]. The advantages of LLIF compared with PLIF or TLIF are avoidance of blood loss from the extradural venous plexus, less invasion of the posterior column and muscle, and possibly faster interbody fusion with a higher fusion rate [11]. During removal of the OYL lesion, the possibility of ossification of the dura mater should be considered [12]. We chose LLIF instead of PLIF or TLIF for interbody fusion procedure for the patient reported herein, to avoid the risk of excess blood loss from the extradural venous plexus and to avoid durotomy during removal of the ossified lesion [13]. Our patient had lumbar OYL at multiple levels and underwent a 2-stage surgery: LLIF and posterior decompression fusion.

## 2. Case Presentation

A 55-year-old man was aware of muscle weakness in both legs and was not able to walk quickly 5 months before surgery. He came to our hospital 3 months before surgery, and his gait disturbance became pronounced requiring the use of a walking stick.

Neurologic examination on admission revealed muscle weakness, with a manual muscle test (MMT) score of 4/4 in the iliopsoas muscle, the quadriceps femoris, and the tibialis anterior and a MMT score of 5/5 in the extensor hallucis brevis, flexor hallucis longus, gastrocnemius, and hamstring muscles. He had a diminished patellar tendon reflex and Achilles tendon reflex in both legs. Although he had numbness in both legs, there was no pain and sensory disturbance in either leg. He reported the feeling of residual urine and reported urinating 10 times each day, and had low urine flow. Preoperative clinical evaluation showed a Japanese Orthopaedic Association (JOA) score of 7/29 (1-1-0, 1-0-1, 1-1-1-0-2-0-0, -3). The Japanese Orthopaedic Association Back Pain Evaluation Questionnaire (JOABPEQ) score was 43 points for lumbar back pain, 8 points for lumbar function, 0 points for walking ability, 22 points for social-life function, and 15 points for mental health. The Zurich Claudication Questionnaire (ZCQ) score was 2.86 for symptom severity and 2.60 for physical function. The Oswestry Disability Index (ODI) was 54.

**Figure 1:**
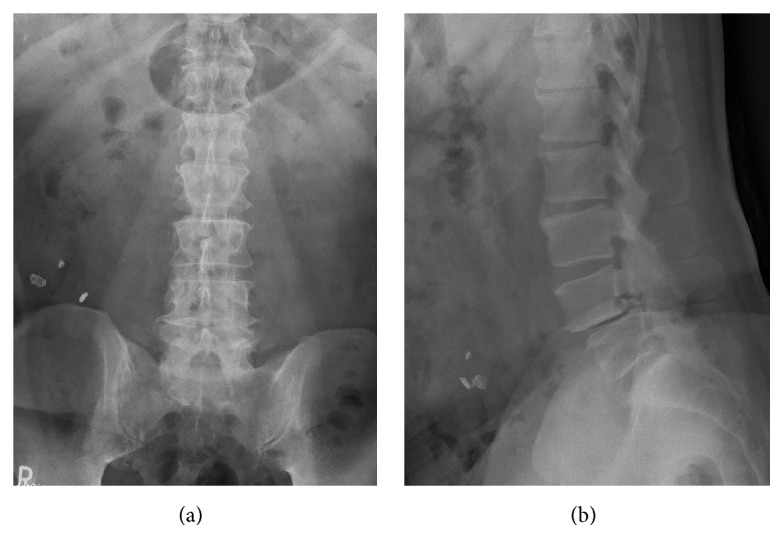
Preoperative radiography of the lumbar spine. Anteroposterior (AP) view (a); lateral view (b). Ossification of the yellow ligament (OYL) is seen at L3/4 and anterior spondylolisthesis of L4 vertebra, with concomitant ossification of the posterior longitudinal ligament.

**Figure 2:**
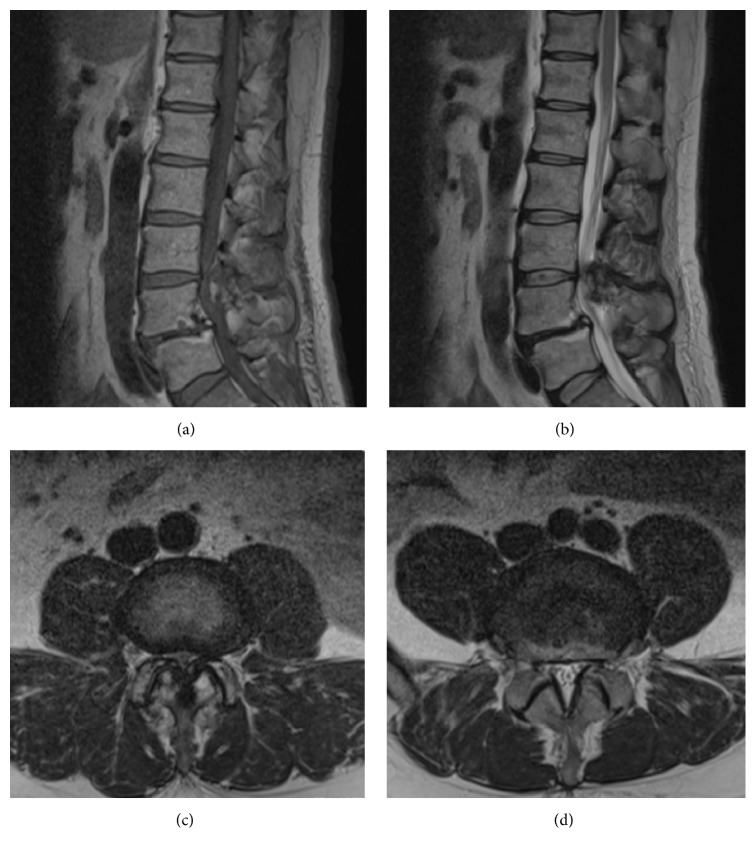
Preoperative magnetic resonance imaging. Sagittal T1-weighted image (a); sagittal T2-weighted image at the midspinal canal (b); axial T2-weighted image at the L3/4 level (c); axial T2-weighted image at the L4/5 level (d). Lumbar canal stenosis is seen at the L3/4 and L4/5 levels, with severe stenosis at L3/4.

**Figure 3:**
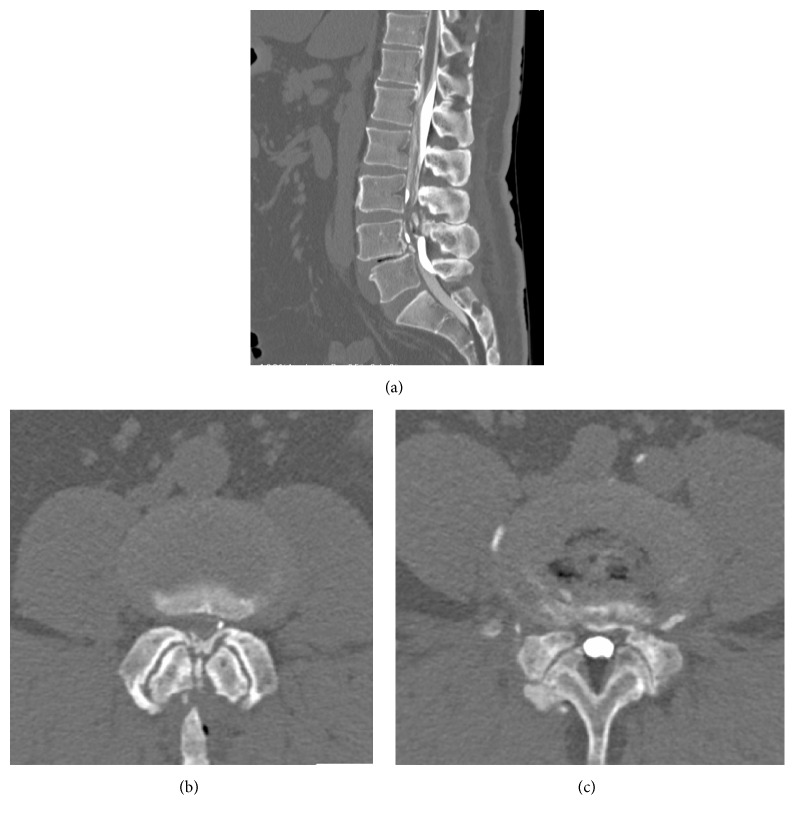
Preoperative computed tomography (CT) myelography. Sagittal image at the midspinal canal (a); axial image at the L3/4 level (b); axial image at the L4/5 level (c). OYL and complete block are seen at the L3/4 level.

Plain radiography of the lumbar spine demonstrated OYL at L3/4 and anterior spondylolisthesis of L4 vertebra, with concomitant ossification of the posterior longitudinal ligament (OPLL) (Figure 1). Magnetic resonance imaging showed lumbar canal stenosis at levels L3/4 and L4/5 and severe stenosis at L3/4 (Figure 2). Computed tomography (CT) myelography revealed OYL and complete block at level L3/4 (Figure 3).

We planned a 2-stage surgery to achieve both safe removal of the OYL lesion and reliable intervertebral fusion and to avoid excess blood loss from manipulating the epidural venous plexus. In this case, there was muscle weakness due to severe canal stenosis by osseous intracanal lesion, so we considered that indirect decompression by ligamentotaxis would not be obtained, and direct decompression would be necessary [14].

We initially performed LLIF at L3/4 and L4/5. We harvested bone from the iliac crest as we approached the disc and used it for a bone autograft in the polyetheretherketone cage (XLIF-cage, Nuvasive, San Diego, CA) used at each disc space. We used a lordotic angle of 10 degrees. This initial operation lasted 216 minutes, and the estimated blood loss was 5 mL. Intraoperative motor-evoked potential monitoring showed no change in amplitude. After the first procedure, numbness in both legs reduced a little, and that was the only change in his neurologic examination. A MMT score of the iliopsoas muscle, the quadriceps femoris, and the tibialis anterior did not change.

**Figure 4:**
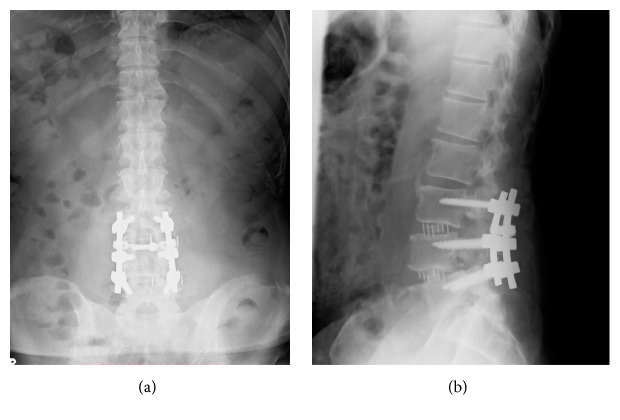
Postoperative radiography of the lumbar spine. AP view (a); lateral view (b). Lateral lumbar interbody fusion of L3/4 and L4/5 was performed at the initial surgery, and posterior decompression fixation using pedicle screws from L3 to L5 was performed at the second surgery.

Seven days after the first procedure, the second operation was performed. We performed posterior decompression under microscopy guidance, after completing posterior fixation with pedicle screws from L3 to L5 (Figure 4). We performed a total L3 laminectomy and bilateral facetectomy of L3/4; the OYL lesion at L3/4 was carefully and completely removed, using a diamond high-speed drill under microscopy guidance. Adhesions between the OYL and the dura mater were carefully peeled away, and no incidental durotomy occurred. The second procedure lasted 233 minutes, and the estimated blood loss was 500 mL. A total of 800 mL of autologous blood storage was prepared preoperatively, and retransfusion was performed after the second surgery.

**Figure 5:**
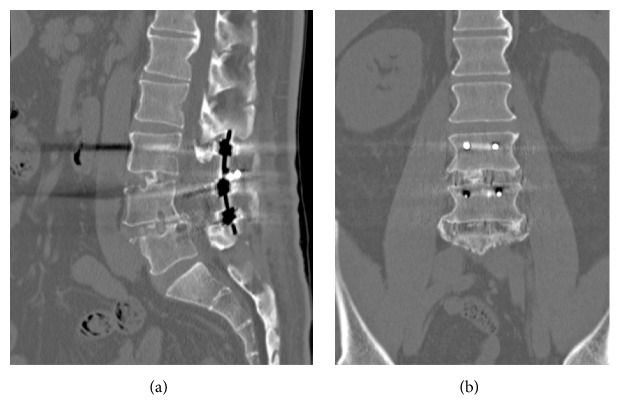
CT 12 months after surgery. Sagittal image at the midspinal canal (a); coronal image at the midvertebral body (b). Bridging bone formation is present at the edge of the cage inserted at the L3/4 and L4/5 intervertebral spaces.

The patient's postoperative course was uneventful, with no perioperative complications. After the second surgery, his leg pain and numbness improved significantly, and he was able to walk without the aid of a stick by the time of hospital discharge. The muscle weakness in both legs, the bladder dysfunction, and the numbness in both legs had completely resolved by the time of his 6-month follow-up. At his 12-month follow-up, CT revealed bridging bone formation at the edge of the cage at the L3/4 and L4/5 intervertebral spaces (Figure 5). Clinical evaluation was performed at 1 year after surgery: the JOA score was 27/29 (3-3-3, 2-2-2, 2-2-2-1-2-1-2, 0), and the JOABPEQ score was 100 points for lumbar back pain, 50 points for lumbar function, 100 points for walking ability, 100 points for social-life function, and 54 points for mental health. The ZCQ score was 1.26 for symptom severity, 1.20 for physical function, and 1.35 for patient satisfaction; the ODI was 12.

## 3. Discussion

Posterior decompression is the most commonly performed surgical procedure for OYL, especially when the lesion is located in the middle thoracic spine, the most common area for OYL to manifest [7]. However, when the lesion occurs in the lower thoracic and lumbar spine, a fusion procedure should be considered to avoid postoperative progression of any residual ossified lesion and to avoid intervertebral instability [7, 15–17]. In our patient, bilateral facetectomy was unavoidable to completely remove the OYL lesion at L3/4; the OPLL at L4/5 was left untouched. The procedures for interbody fusion, such as intervertebral disc dissection, are difficult when adhesions exist between the ossified lesion and the thecal sac or when ossification of the dura mater is present [6, 12].

The technique of LLIF is relatively new, first reported in the 1990s, and its use spread widely in Western countries in the early 2000s [8, 18]. The procedure was introduced to Japan in 2013 and was rapidly adopted because of its advantages: better coronal- and sagittal-alignment correction for degenerative scoliosis, the usefulness of indirect decompression for central- and foraminal-canal stenosis, and its low intraoperative blood loss compared with posterior intervertebral fusion procedures [10, 19]. The LLIF technique allows increased surface contact area between the cage and the bone, and Berjano et al. reported a high fusion rate and satisfactory clinical outcomes [11]. Another important advantage of LLIF is less surgical blood loss compared with PLIF or TLIF [13].

To the best of our knowledge, this is the first documented report of lumbar OYL treated with LLIF and posterior decompression fusion. We performed a 2-stage surgery with LLIF and posterior decompression and fusion because of concomitant OYL at L3/4, OPLL at L4/5, anterior spondylolisthesis of L4 vertebra, and anticipated adhesions between the thecal sac and the ossified lesion. Our total blood loss was satisfactory at 500 mL, and allogeneic blood transfusion was unnecessary. Furthermore, we safely and successfully performed complete resection of the OYL lesion and achieved adequate decompression fusion. The patient experienced no complications.

## 4. Conclusion

Two-stage LLIF and posterior decompression fusion is a good surgical option for lumbar OYL, especially in a patient in whom facetectomy for spinal-cord decompression is inevitable. The advantages of this procedure are less risk of injury to the nerve roots and thecal sac and less blood loss during surgery since the epidural venous plexus may be left untouched.
